# Integrated experimental and computational analysis reveals amoxicillin binding dynamics to PBP1a in Staphylococcus aureus

**DOI:** 10.1038/s41598-025-07626-x

**Published:** 2025-06-25

**Authors:** Seifeldin Elabed, Mariam Ali, Shrouk Hanafy, Sohila Mostafa, Momen Mamdouh, Ayman Meselhi

**Affiliations:** Medical Biophysics Division, Department of Physics, Faculty of Science, Helwan university, Cairo, 11795 Egypt

**Keywords:** Nanobiotechnology, Antibiotics, Computational models

## Abstract

Methicillin-resistant *Staphylococcus aureus* (MRSA) represents a significant global health challenge due to acquired resistance mechanisms, primarily involving penicillin-binding protein 2a (PBP2a), necessitating novel therapeutic strategies. This study explores the potential of amoxicillin-conjugated magnetic nanoparticles (Amox-MNPs) as a means to overcome resistance by targeting the alternative essential protein, PBP1a. Fe₃O₄@SiO₂ core-shell MNPs were synthesized via controlled co-precipitation followed by a silica coating using the Stöber method, and subsequently conjugated with amoxicillin. Physicochemical characterization confirmed nanoparticle formation and successful conjugation. In vitro antibacterial assays against *S. aureus* ATCC 43,300 (MRSA) revealed that Amox-MNPs exhibited a mean inhibition zone diameter of 26.0 ± 0.82 mm, approximately double that of free amoxicillin (13.5 ± 1.12 mm) at equivalent concentrations (*p* < 0.05), indicating significantly enhanced antibacterial efficacy. Integrated computational modeling, including molecular docking and dynamics simulations, elucidated the favorable binding (−8.64 kcal/mol docking score;−32.65 kcal/mol MM-PBSA energy) and stable interaction dynamics between amoxicillin and PBP1a, identifying key stabilizing residues. These findings highlight the potential of MNP-mediated delivery to enhance amoxicillin’s efficacy against MRSA by targeting PBP1a, offering a promising preclinical strategy requiring further validation in animal models for combating resistant bacterial infections.

**Supplementary Information:**

The online version contains supplementary material available at 10.1038/s41598-025-07626-x.

## Introduction

Methicillin-resistant *Staphylococcus aureus* (MRSA) poses a growing global health threat, accounting for around 94,000 invasive infections annually in the U.S. alone, with significantly higher mortality rates than methicillin-susceptible strains^1^. MRSA’s resilience stems from its remarkable genetic adaptability, enabling rapid acquisition of resistance via horizontal gene transfer and proliferation in both healthcare and community settings—including reservoirs in livestock and companion animals^2^.

The primary resistance mechanism in MRSA involves the *mecA*-encoded penicillin-binding protein 2a (PBP2a), which exhibits low affinity for β-lactam antibiotics, allowing continued peptidoglycan cross-linking during cell wall synthesis despite antibiotic presence^2,3^. Resistance is further compounded by the integration of mobile genetic elements such as staphylococcal cassette chromosome *mec* (SCC*mec*), production of β-lactamases, and alterations to cell wall structure and permeability^2,4^.

In response to these multidrug resistance mechanisms, nanoparticle-based drug delivery systems (NDDS) have emerged as a promising strategy^5^. Nanomaterials offer several advantages: they enhance local drug concentrations at infection sites, penetrate bacterial barriers such as biofilms, and shield antibiotics from enzymatic degradation^6^. Among these, superparamagnetic iron oxide nanoparticles (Fe₃O₄ MNPs) stand out due to their biocompatibility, ease of functionalization, and potential for magnetically guided targeting^7^. Uncoated Fe₃O₄ cores further improve drug-loading efficiency and magnetic responsiveness^7^.

Beyond delivery platforms, nanomaterials can possess intrinsic antimicrobial activity. For example, nanoemulsions disrupt bacterial membranes and interfere with cellular functions in drug-resistant ESKAPE pathogens^6^. Biogenic metal nanoparticles—such as silver or zinc-based—exert potent bactericidal and antibiofilm effects through ion release and reactive oxygen species (ROS) generation^8^. Use of silver nanoparticles synthesized from silver-tolerant bacteria further enhances cellular uptake and efficacy^9^. Notably, applications from aquaculture (e.g., nanoparticles against acute hepatopancreatic necrosis disease) illustrate the broader utility of nanotechnology in infectious disease control^10^. Collectively, these findings underscore the synergistic potential of combining functional nanomaterials like Fe₃O₄ with antibiotics to overcome resistance.

While PBP2a confers β-lactam resistance, other penicillin-binding proteins (PBPs) remain viable targets. PBP1a, a high-molecular-weight class A PBP, is essential for septal peptidoglycan synthesis and cross-linking during cell division. Inhibiting its transpeptidase activity or PASTA domain function impairs cell viability, marking it as a strategic antimicrobial target^11^. Importantly, PBP1a retains susceptibility to certain β-lactams, including ceftaroline, which exhibits sub-micromolar Ki values across diverse MRSA strains^12^. This suggests that PBP1a provides a functionally accessible β-lactam-binding site, even in the presence of PBP2a.

Despite advances in NDDS and the known vulnerability of PBP1a, the use of amoxicillin—a widely used β-lactam—conjugated to Fe₃O₄ MNPs for MRSA treatment remains largely unexplored. Prior work has shown antimicrobial effects of amoxicillin conjugated to gold nanoparticles^13^, and the efficacy of MNPs in antimicrobial applications^7^, but the specific interaction between amoxicillin-loaded Fe₃O₄ MNPs and PBP1a has not been characterized.

This study addresses this gap by synthesizing and characterizing amoxicillin-conjugated Fe₃O₄@SiO₂ MNPs and evaluating their interaction with MRSA’s PBP1a via molecular docking and molecular dynamics simulations. This combined experimental-computational approach enables atomistic insight into the binding dynamics between amoxicillin and PBP1a, while also assessing the delivery potential of Fe₃O₄ MNPs. Ultimately, the study aims to provide a scientific rationale for developing amoxicillin-MNP formulations as a novel strategy to circumvent PBP2a-mediated resistance by targeting alternative PBPs, contributing to next-generation antimicrobial design.

## Materials and methods

### Nanoparticle physicochemical properties

#### Materials

All chemicals and reagents used in this study were of analytical grade and utilized without further purification. Ferric chloride hexahydrate (FeCl₃·6 H₂O), ferrous chloride tetrahydrate (FeCl₂·4 H₂O), tetraethyl orthosilicate (TEOS), ammonium hydroxide solution (28–30%), ethanol (≥ 70%), and phosphate-buffered saline (PBS, pH 7.4) were purchased from Sigma-Aldrich (USA). Amoxicillin trihydrate was obtained from a certified pharmaceutical supplier and used as the model antibiotic. Deionized water (resistivity ≥ 18 MΩ·cm) was used throughout all preparation and washing steps. Staphylococcus aureus ATCC 43,300 was acquired from the American Type Culture Collection (ATCC) and cultured on Mueller-Hinton agar (MHA) plates according to CLSI guidelines. All solutions and media were prepared using sterile techniques. Analytical-grade reagents were employed for UV–Visible spectrophotometry and dynamic light scattering (DLS), while microscopy-grade grids and solvents were used for transmission electron microscopy (TEM) imaging. All materials and consumables adhered to standard laboratory biosafety and quality control practices.

#### Synthesis of fe₃o₄@sio₂ nanoparticles

In this study, magnetite (Fe₃O₄) nanoparticles were synthesized via a co-precipitation method in which ferric chloride hexahydrate (FeCl₃·6 H₂O) and ferrous chloride tetrahydrate (FeCl₂·4 H₂O) were combined in a 2∶1 molar ratio under vigorous stirring at 25 °C. Ammonium hydroxide (28–30%) was added dropwise until the pH reached 10–11, resulting in the instantaneous formation of a black precipitate. The precipitate was repeatedly washed with 70% ethanol, which effectively removes hydrophobic contaminants and any adsorbed organic residues, followed by rinsing with deionized water to neutralize and eliminate soluble ionic species such as chloride ions. These repeated washing steps are essential for ensuring the purity and stability of the Fe₃O₄ cores prior to further functionalization. The cleaned precipitate was then vacuum-dried overnight at 60 °C and subsequently dispersed in a 3∶1 ethanol–water (v/v) mixture containing 10% (v/v) tetraethyl orthosilicate (TEOS) at pH 9.0. Heating this dispersion to 90 °C for 6 h induced the Stöber process to uniformly coat the magnetite cores with a silica shell, yielding Fe₃O₄@SiO₂ nanoparticles^7,14–16^.

#### Amoxicillin conjugation

For antibiotic conjugation, silica-coated nanoparticles (10 mg) were resuspended in 10 mL phosphate-buffered saline (PBS, pH 7.4) containing amoxicillin trihydrate at a concentration calculated such that each experimental plate ultimately received 30 µg of amoxicillin. The suspension was incubated at 25 °C with gentle agitation for 24 h, allowing the antibiotic to adsorb onto the silica surfaces primarily through silanol–amine interactions. An equivalent free amoxicillin solution (30 µg per plate) was prepared as a control. After incubation, the nanoparticles were separated by centrifugation at 10,000 rpm for 20 min, and the supernatant was analyzed using UV–Visible spectroscopy to confirm complete drug loading. The amoxicillin-conjugated nanoparticles were washed with PBS, resuspended to the initial volume, and stored at 4 °C until use^17,18^.

#### Characterization of nanoparticles

Characterization of the nanoparticle-antibiotic complexes was carried out to ensure reproducibility and colloidal stability. The hydrodynamic diameter and polydispersity index of both free MNPs and amoxicillin-conjugated MNPs were measured by dynamic light scattering (DLS) on a Malvern Zetasizer Nano ZS at 25 °C, with samples diluted fivefold in deionized water and each measurement repeated in triplicate. Zeta potential measurements under identical conditions provided insights into the surface charge and colloidal stability of the conjugates. The morphology and core-size distribution were examined by transmission electron microscopy (TEM) using a JEOL JEM−2100 F operating at 200 kV, while UV–Visible spectrophotometry (200–600 nm) was employed to monitor absorbance shifts indicative of successful amoxicillin conjugation. Recent studies emphasize the significance of these analytical methods in verifying nanoparticle stability and optimizing their interaction with biological systems^19,20,21^.

#### Antibacterial activity evaluation

The antibacterial activity of the synthesized nanoparticles and controls was assessed against *Staphylococcus aureus* ATCC 43,300 using the agar well diffusion method on Mueller–Hinton agar (MHA) plates, following Clinical and Laboratory Standards Institute (CLSI) M02 guidelines^22,23,24^. A bacterial suspension was prepared in sterile PBS (pH 7.4) and adjusted to a turbidity equivalent to a 0.5 McFarland standard (~ 1 × 10⁸ CFU/mL)^25^. This suspension was uniformly swabbed onto the surface of the MHA plates. Wells (6 mm diameter) were aseptically punched into the agar. Each well was loaded with a sample volume delivering free amoxicillin (30 µg/mL) and amoxicillin-MNPs (30 µg/mL equivalent). Four plates were prepared per plate for each sample type. The plates were incubated at 37 °C for 24 h. After incubation, the diameter of the inhibition zones around each well was measured in millimeters (mm) using a caliper. Measurements for each condition were performed in triplicate. All statistical analyses were implemented in R, employing the latest version of the tidyplots package for data visualization and validation of results^21,26^. The Wilcoxon Rank-Sum Test was used to compare the diameters of the inhibition zones between different treatment groups. A permutation test (10,000 permutations, random seed set to 123) was also conducted.

### Computational methodology

#### Structural identification

The primary amino acid sequence of *Staphylococcus aureus* PBP1a was obtained from the UniProt database (A0A0H2WVW5|A0A0H2WVW5_STAAC). Conserved domains and active-site motifs were identified using InterProScan 5, which integrates multiple signature databases (e.g., Pfam, SMART)^27^.

#### Molecular Docking

The three-dimensional structure of amoxicillin (PubChem CID 33613) was retrieved from the PubChem database. The structure was converted to PDB format using Open Babel^28^, and its energy was minimized using the MMFF94 force field to generate a low-energy conformation^29^. AutoDockTools 4 was used to assign Gasteiger partial charges and define rotatable bonds for the ligand^30^. The crystal structure of PBP1a (PDB ID: 5TRO) was obtained from the Protein Data Bank^31^. Preparation involved removing crystallographic water molecules, adding polar hydrogens, and assigning Kollman charges using AutoDockTools^30^. A grid box of 24 × 24 × 24 Å³ was defined, centered on the catalytic region (including Ser368–Thr516) identified through conserved active-site residue analysis from Structural Identification Stage. Rigid-body docking was performed using AutoDock Vina, with an exhaustiveness parameter set to 16, generating 20 binding modes^32^. Rigid docking was deemed appropriate based on the typically limited backbone flexibility observed in class A PBPs, where ligand binding often induces only minor loop movements (≤ 1 Å)^33^. The top five poses, ranked by binding affinity, were retained, and the conformation with the best score (kcal/mol) was selected for subsequent analysis. Protein–ligand interactions for this pose were characterized in 3D using the Protein-Ligand Interaction Profiler (PLIP)^34^ and visualized in 2D using Discovery Studio 2025^35^.

#### Molecular dynamics simulations and MM-PBSA analysis

All-atom molecular dynamics (MD) simulations were performed using GROMACS version 2022.4^36,37^. The CHARMM36 force field was applied to the protein^38^, while CHARMM General Force Field (CGenFF) parameters were used for amoxicillin^39^. The protein-ligand complex was solvated within a cubic box using the TIP3P water model, maintaining a 10 Å buffer distance from the box edges^40^. The system was neutralized with Na⁺ and Cl⁻ ions to achieve an ionic strength of 0.15 M. Energy minimization was conducted using the steepest-descent algorithm until the maximum force dropped below 1000 kJ⋅mol−1⋅nm−1. The system was then equilibrated through two stages: 100 ps in the NVT ensemble (constant volume, 300 K using the velocity-rescaling thermostat^41^) followed by 100 ps in the NPT ensemble (constant pressure, 1 atm using the Parrinello–Rahman barostat^42^). A production MD run of 100 ns was executed with a 2 fs time step. Long-range electrostatic interactions were handled using the Particle Mesh Ewald (PME) method with a 10 Å cutoff^43^. Trajectory frames were saved every 10 ps. System equilibration and complex stability were assessed by analyzing root-mean-square deviation (RMSD), root-mean-square fluctuation (RMSF), radius of gyration, and hydrogen bonds using tools integrated within GROMACS^36,37^. Equilibration was generally observed after approximately 10 ns.

End-state binding free energies ($\:{\varDelta\:G}_{bind}$) were calculated using the Molecular Mechanics Poisson-Boltzmann Surface Area (MM-PBSA) method as implemented in the g_mmpbsa tool^44^. The binding free energy ($\:{\varDelta\:G}_{bind}$) was calculated using the equation:

$$\:{\varDelta\:G}_{bind}\:=\:{G}_{complex}-\:({G}_{protein}+{G}_{ligand})$$

where $\:\:{G}_{complex}$,$\:{G}_{protein}$,$\:{G}_{ligand}$ and are the free energies of the complex, protein, and ligand, respectively.

For this calculation, 200 frames were extracted evenly from the full portion of the 100 ns trajectory with 50 frame intervals. Van der Waals, electrostatic, polar solvation, and non-polar solvation energy components were computed for the complex, receptor, and ligand states, allowing for the calculation of $\:{\varDelta\:G}_{bind}$ via the standard MM-PBSA equation [44].

Visualization of trajectories and plotting of energy components and structural analyses were performed using seaborn^45^, Plotly^46^, and Matplotlib^47^ to generate publication-quality figures.

## Results and discussion

### Nanoparticle physicochemical properties

#### Surface charge and stability

**Fig. 1:**
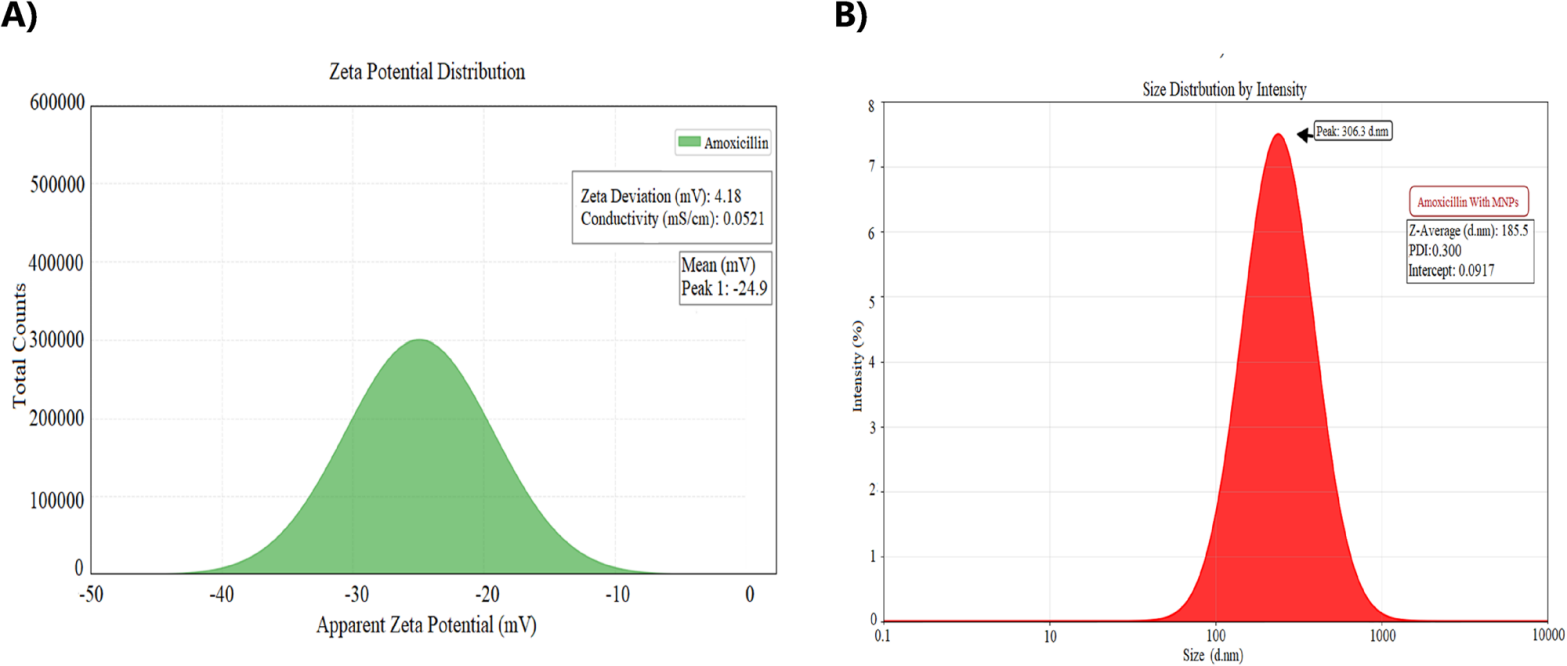
(**A**) Zeta-potential distribution of amoxicillin–MNPs (mean − 24.9 ± 4.18 mV), demonstrating strong negative surface charge contributing to colloidal stability. (**B**) DLS size distribution profile (Z-average = 335.5 nm, PDI = 0.30), reflecting the hydrodynamic size and moderate polydispersity of the nanoparticles in suspension.

The synthesized amoxicillin-conjugated magnetic nanoparticles (amoxicillin–MNPs) were characterized to understand their physicochemical properties relevant to colloidal stability and biological interaction. Zeta potential analysis revealed a mean value of−24.9 ± 4.18 mV (Fig. 1A), indicating a strong negative surface charge at physiological pH. This magnitude of surface charge generally imparts sufficient electrostatic repulsion between nanoparticles to maintain colloidal stability and prevent significant aggregation over time^48,49^. While absolute zeta potentials exceeding 30 mV are often cited as thresholds for “good” stability, values around−25 mV, as observed here, are still considered effective in promoting particle dispersity, particularly when supported by low ionic conductivity (measured at 0.052 mS/cm), which suggests a robust electrical double layer less susceptible to salt-induced coagulation^48,50^.

A pertinent question arises regarding the interaction between these negatively charged nanoparticles and the predominantly negatively charged surface of bacterial cell walls^51^. While electrostatic repulsion might seem inhibitory, several factors can facilitate nanoparticle-bacteria interactions. Firstly, bacterial surfaces possess localized microdomains with varying charge distributions, potentially including positively charged patches (e.g., associated with proteins or peptidoglycan modifications) that can serve as initial docking sites for anionic nanoparticles^52^. Secondly, non-electrostatic forces, such as van der Waals interactions, hydrophobic interactions, and specific ligand-receptor binding (if applicable), can play a significant role, especially at close proximity^53^. Furthermore, the high surface area-to-volume ratio of nanoparticles leads to a high concentration gradient of the conjugated antibiotic (amoxicillin) near the bacterial surface, potentially overcoming repulsive forces and driving interaction^54^. The observed potent antibacterial effect strongly suggests that effective interaction and subsequent drug delivery do occur, warranting further investigation into the precise binding mechanisms.

#### Size distribution and polydispersity

**Fig. 2:**
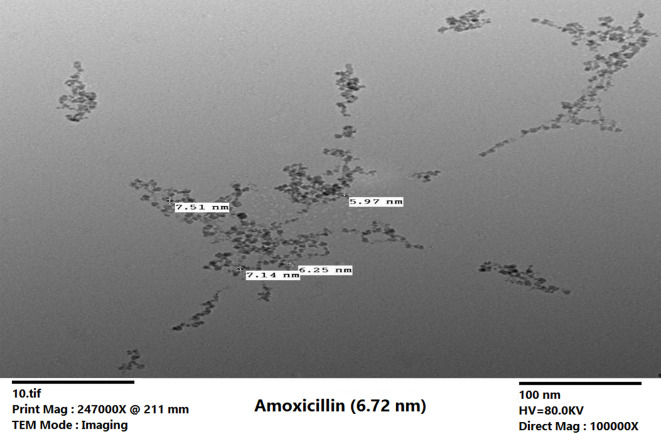
Representative TEM micrographs of amoxicillin–MNPs, revealing spherical iron-oxide cores (6–8 nm diameter) and the formation of chain-like aggregates characteristic of magnetic nanoparticle interactions in the dried state.

Dynamic light scattering (DLS) measurements indicated a Z-average hydrodynamic diameter of 335.5 nm with a primary peak observed at 306.3 nm (Fig. 1B). The polydispersity index (PDI) was calculated to be 0.30. PDI values up to 0.3 are generally considered acceptable for nanoparticle drug delivery systems, signifying a moderate degree of heterogeneity in particle size distribution that typically does not compromise therapeutic performance^55^. Transmission electron microscopy (TEM) imaging provided complementary information, revealing the core size of the iron-oxide nanoparticles to be approximately 6–8 nm (Fig. 2). TEM images also showed the characteristic spherical morphology and the tendency of magnetic nanoparticles to form chain-like aggregates in the dried state, likely due to magnetic dipole interactions^56^. The significant difference between the TEM core size and the DLS hydrodynamic diameter is expected. The hydrodynamic diameter measured by DLS reflects the size of the particle as it moves in suspension, encompassing not only the inorganic core but also the silica shell, the conjugated amoxicillin layer, and a surrounding hydration shell (hydrodynamic layer), all of which contribute to the larger apparent size in the aqueous environment^57^.

#### Molecular conjugation confirmed by UV–Vis spectroscopy

**Fig. 3:**
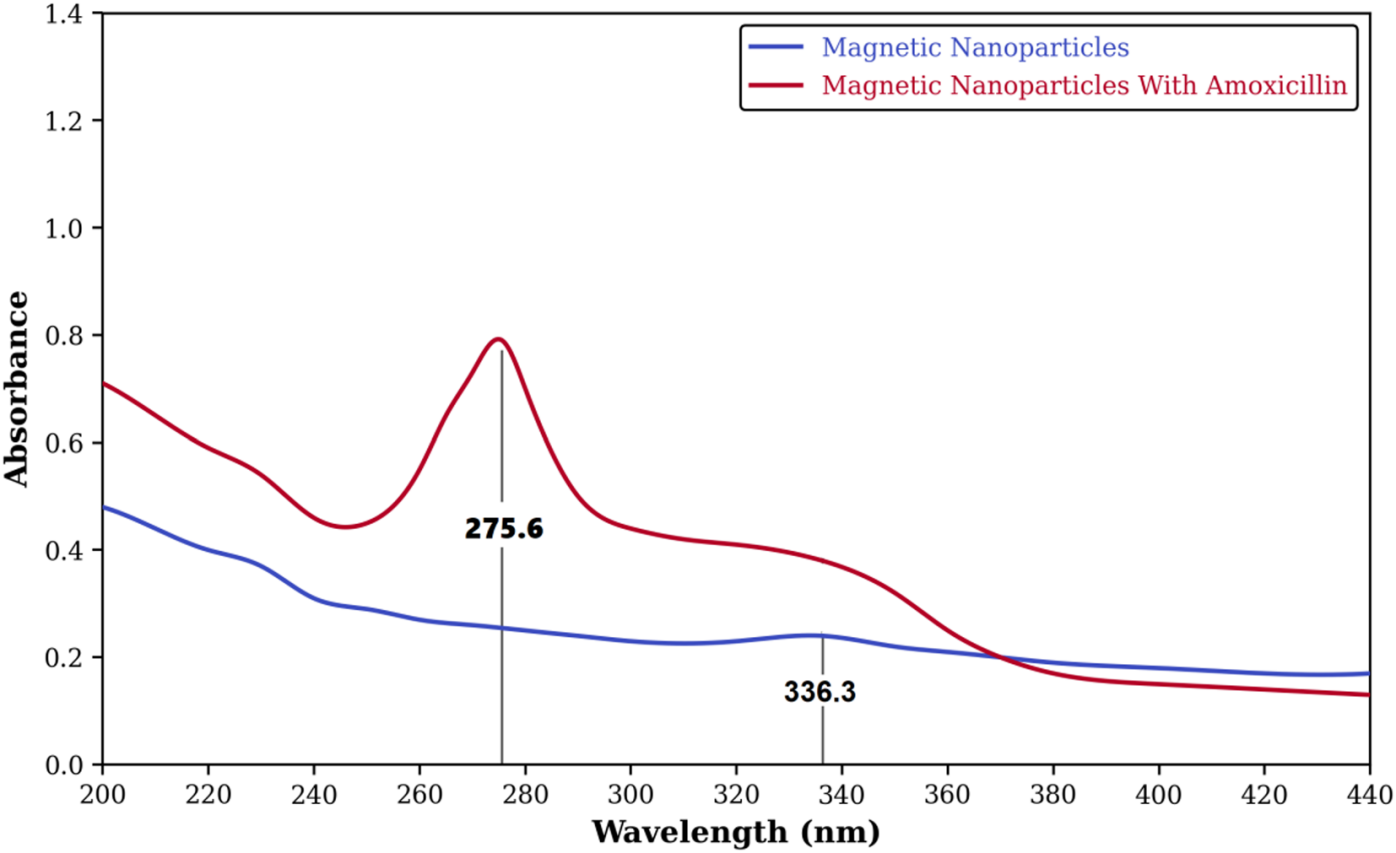
Comparative UV–Vis spectra showing unmodified MNPs (blue baseline) and amoxicillin–MNPs (red line). The bathochromic shift and hyperchromic effect observed around 275.6 nm for amoxicillin–MNPs confirm successful antibiotic conjugation.

The successful conjugation of amoxicillin to the surface of the silica-coated MNPs was confirmed using UV–Vis spectroscopy (Fig. 3). Free amoxicillin in aqueous solution exhibits characteristic absorbance maxima around 228 nm and 278 nm^58^. In contrast, the spectrum of the amoxicillin–MNPs showed distinct changes compared to unmodified MNPs (used as a baseline). Specifically, a bathochromic (red) shift of the peak to approximately 275.6 nm was observed, accompanied by a hyperchromic effect (increased absorbance intensity) in the 220–300 nm region. These spectral modifications are indicative of the successful covalent attachment or strong adsorption of the amoxicillin molecule, containing the β-lactam aromatic chromophore, onto the nanoparticle surface^58,59^.

#### Analysis of TEM micrographs

Transmission electron microscopy (TEM) measurements, conducted in the dry state, revealed a core diameter of approximately 6–8 nm for the amoxicillin-conjugated magnetic nanoparticles, as shown in Fig. 2. In contrast, dynamic light scattering (DLS) performed in aqueous suspension yielded an average hydrodynamic diameter of around 306 nm.

This discrepancy is primarily attributed to the additional volume contributed by the silica coating and the conjugated amoxicillin molecules. Moreover, DLS measurements inherently include the surrounding hydration shell that is absent in TEM imaging. Together, these factors account for the significantly larger effective size observed in solution compared to the dry-state core size.

Further evidence of size discrepancy is provided by the TEM micrographs, which revealed the formation of chain-like aggregates—a common phenomenon for magnetic nanoparticles due to their intrinsic magnetic dipole interactions. These aggregates, although visible in the dry state, are more pronounced in solution where they further increase the measured hydrodynamic diameter. The combination of the hydration layer, the volume of the surface coatings, and the tendency of the nanoparticles to form loose aggregates ensures that the DLS data accurately reflect the behavior of the nanoparticles in their operational environment, without compromising their antibacterial functionality.

#### Antibacterial efficacy and mechanistic insights

**Fig. 4:**
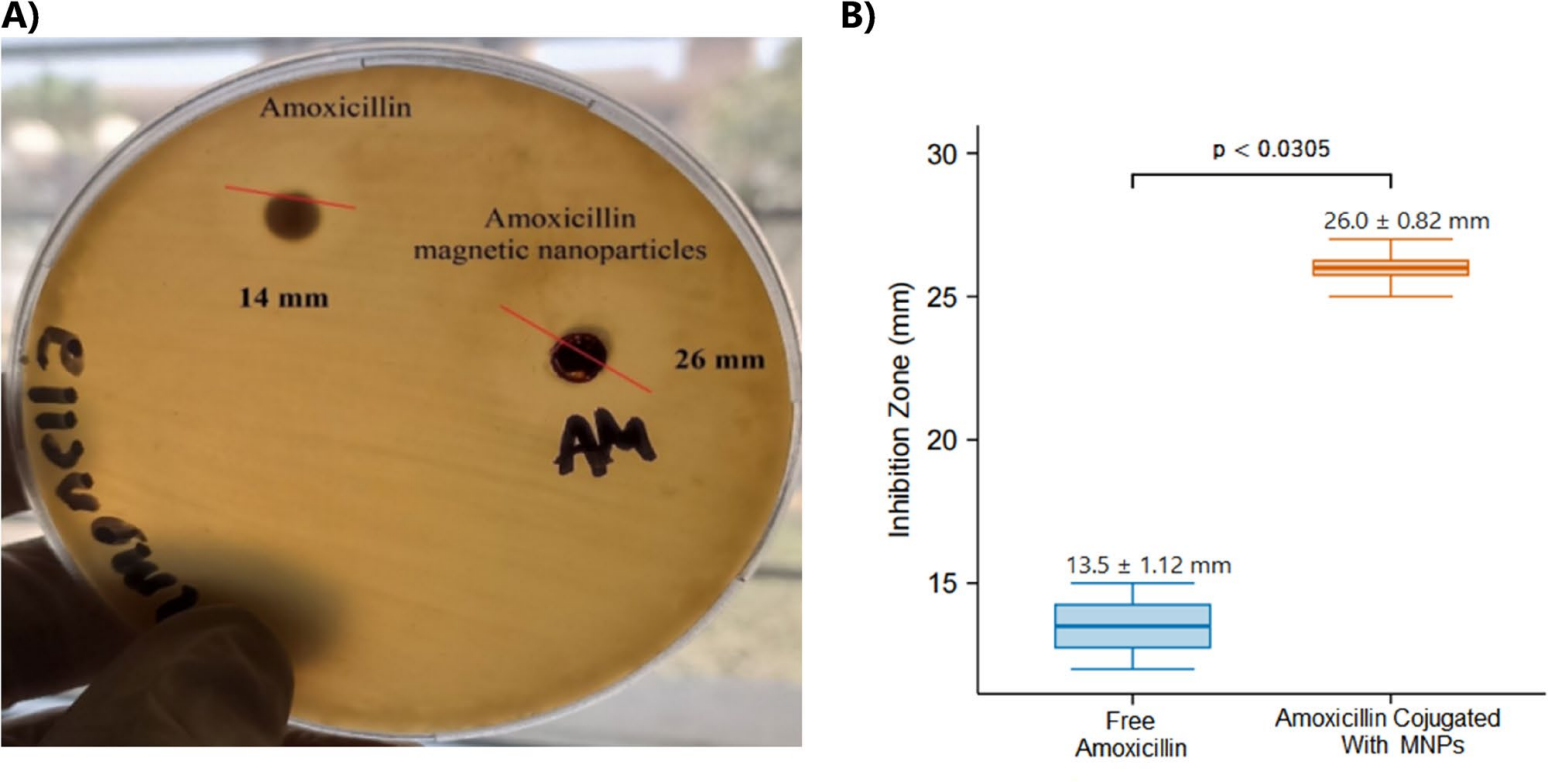
(**A**) Representative image from an agar diffusion assay plate comparing the inhibition zones produced by amoxicillin-conjugated MNPs (e.g., ~ 26 mm) and free amoxicillin (e.g., ~ 14 mm) against *Staphylococcus aureus*. (**B**) Bar graph summarizing the mean inhibition zone diameters (mean ± standard deviation, *n* = 4) for amoxicillin-conjugated MNPs versus free amoxicillin, highlighting the statistically significant difference (*p* < 0.05).

The therapeutic potential of the amoxicillin–MNPs was evaluated against Staphylococcus aureus using agar diffusion assays. Four independent replicates, using aliquots from the same characterized nanoparticle batch, were performed. The results, with a representative image from the agar diffusion assay shown in Fig. 4A, demonstrated a significantly enhanced antibacterial effect for the conjugated nanoparticles compared to the free drug. The mean inhibition zone diameter for amoxicillin–MNPs was 26.0 ± 0.82 mm, nearly double that observed for free amoxicillin at an equivalent concentration (13.5 ± 1.12 mm) (Fig. 4B). The statistical significance of this enhancement was confirmed using the nonparametric Wilcoxon Rank-Sum test (*p* = 0.028). Further validation through permutation testing (10,000 permutations) yielded a comparable significance level (*p* = 0.0305). A bootstrap 95% confidence interval for the mean difference in inhibition zones (–13.75 mm to−11.25 mm) reinforces the robustness and substantial magnitude of the observed effect. Detailed statistical results are summarized in Table 1.

**Table 1:** Comparative statistical analysis of Inhibition zone diameters for amoxicillin–MNPs versus free amoxicillin against *Staphylococcus aureus*.

Test	Test Statistic/Estimate	*p*-value	95% CI
Wilcoxon rank-sum test	W = 0	0.0294	—
Permutation test	Mean difference = −12.25	0.0305	—
Bootstrap (mean diff)	Mean difference = −12.25	—	[−13.75, −11.25]

Mechanistically, this enhanced efficacy can be attributed to several nanoparticle-mediated advantages. The high surface-to-volume ratio inherent to nanomaterials allows the MNPs to act as carriers that concentrate amoxicillin directly at the bacterial cell surface, thereby increasing the local drug concentration available for interaction with penicillin-binding proteins (PBPs) in the bacterial cell wall^54,60^. Furthermore, the protective silica shell encapsulating the amoxicillin molecule likely plays a crucial role in shielding the labile β-lactam ring from degradation by extracellular β-lactamases, enzymes commonly produced by resistant bacterial strains like S. aureus. This protection preserves the antibiotic’s structural integrity and activity until it reaches its target site^61^. The combination of localized delivery and protection against enzymatic inactivation likely underpins the superior performance of the amoxicillin–MNPs compared to free amoxicillin.

### In Silico study

#### Structure-Function analysis of PBP1a (5TRO): implications for antimicrobial activity

**Fig. 5:**
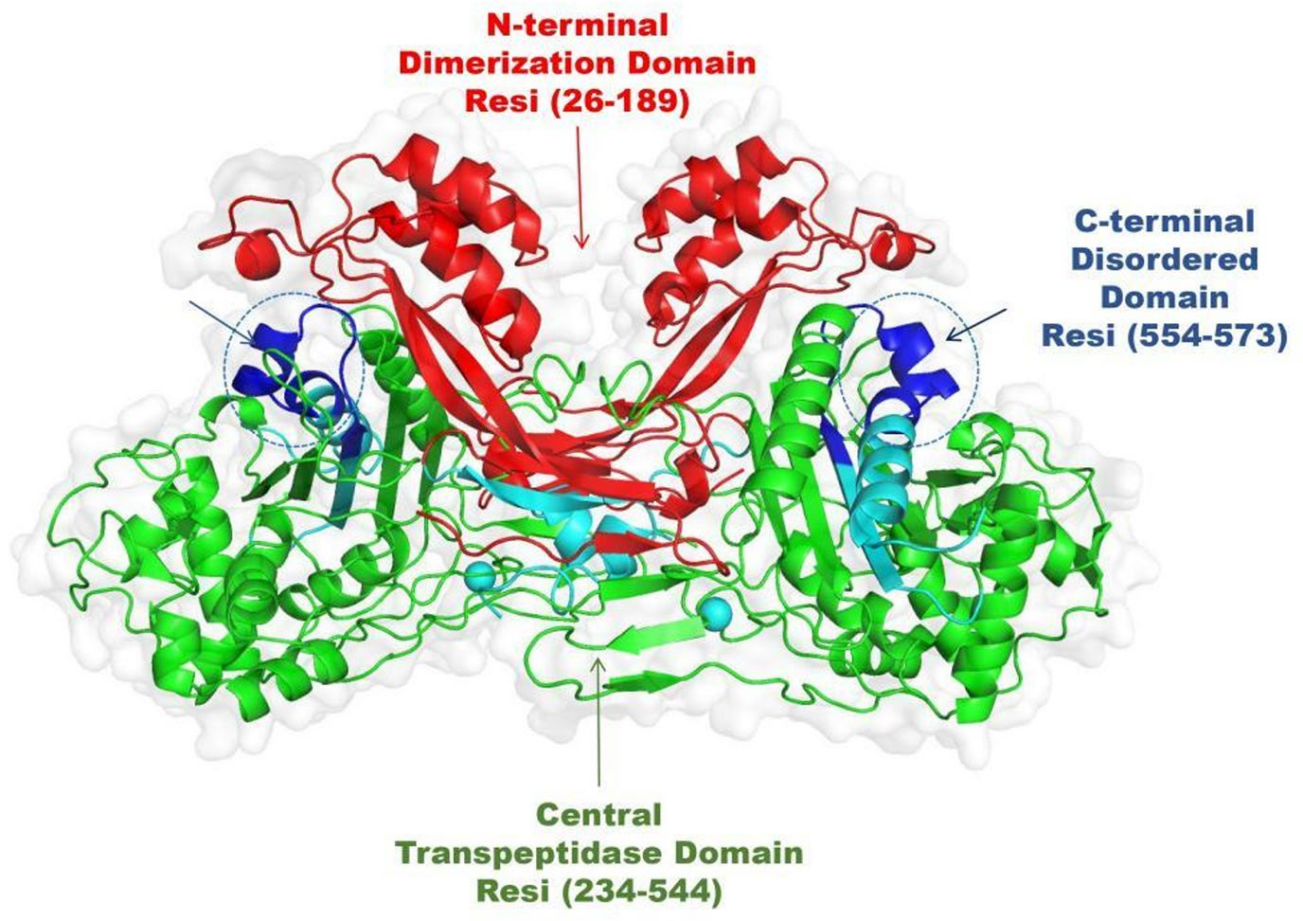
Structural organization and domain architecture of PBP1a (5TRO).

The structural analysis of Penicillin Binding Protein 1 (PBP1a) reveals a domain architecture, illustrated in Fig. 5, that underpins its functional roles in bacterial cell wall synthesis. InterProScan data identify a tripartite domain organization within the PBP1a protein, which spans a variety of essential functional regions. At the N-terminus, spanning residues 26–189, the penicillin-binding protein dimerization domain (PF03717) is responsible for mediating protein-protein interactions crucial for maintaining the quaternary structure of the enzyme. Supporting this observation, Gene3D annotations correspond to matches G3DSA:3.90.1310.10 (residues 29–175) and G3DSA:3.30.70.2110 (residues 47–130), further supporting the presence of a dimerization domain. Moving to the central region, residues 234–544 host the catalytic transpeptidase domain (PF00905), a component for cross-linking peptidoglycan strands during bacterial cell wall biosynthesis. This region aligns with the SUPERFAMILY match SSF56601 (residues 160–555), an association that suggests the domain’s involvement in numerous metabolic pathways, with references from MetaCyc and Reactome indicating its role in cell wall synthesis. The C-terminal domain (residues 554–573) was identified as a disordered segment by MobiDBLite, a feature often linked to structural flexibility. Such regions frequently play roles in mediating transient interactions with other components of the cell wall synthesis machinery or regulatory molecules. Using the PANTHER classification system, PBP1a is categorized within the PTHR30627 family (residues 8–557), which includes proteins involved in bacterial cell wall biosynthesis. This classification is reinforced by FunFam matches, including G3DSA:3.30.70.2110:FF:000001 (residues 47–130) and G3DSA:3.40.710.10:FF:000026 (residues 205–552), which further support its role as a peptidoglycan synthetase. Moreover, the association with the Gene Ontology term GO:0008658 (penicillin binding), as evidenced by the SUPERFAMILY match SSF56519 (residues 27–207), indicates PBP1a’s ability to interact with β-lactam antibiotics, supporting its role in cell wall formation.

#### Molecular Docking analysis of amoxicillin and penicillin binding protein 1 (PBP1a)

Molecular docking was employed to assess the binding of ligand UNL to Penicillin Binding Protein 1a (PBP1a), using the crystal structure from PDB entry [5TRO, Chain B]. The top-scoring pose, obtained using AutoDock Vina, demonstrated a predicted binding affinity of − 8.640 kcal/mol, indicating a favorable interaction. Additional poses with slightly lower affinities (− 8.089 to − 7.366 kcal/mol; Table 2) suggest possible alternative binding orientations.

**Table 2:** Predicted binding affinities and RMSD values for the top Docking poses of ligand UNL with PBP1a (5TRO).

Pose	Binding Affinity (kcal/mol)	RMSD Lower Bound (Å)	RMSD Upper Bound (Å)
1	−8.64	0	0
2	−8.089	17.283	21.628
3	−7.873	16.156	17.88
4	−7.632	16.45	20.346
5	−7.512	15.104	17.741
6	−7.366	16.599	18.853

**Fig. 6:**
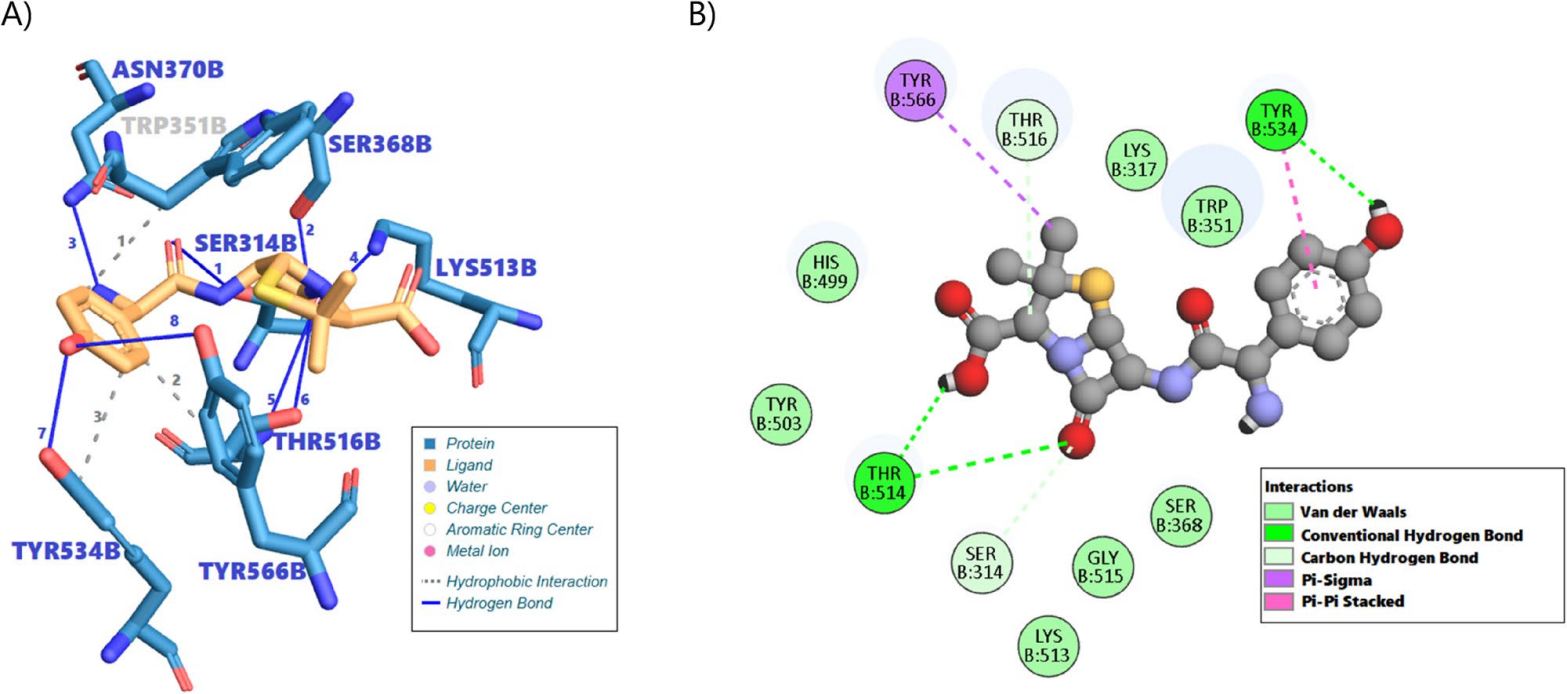
Predicted molecular interactions between Amoxicillin and PBP1a (PDB: 5TRO, Chain B) from molecular docking. (**A**) 3D representation illustrating the binding of Amoxicillin within the PBP1a active site. (**B**) 2D schematic diagram depicting the detailed network of non-covalent interactions.

This analysis revealed a network of specific contacts stabilizing the ligand UNL within the binding pocket of PBP1a chain B (Fig. 6A). Hydrophobic interactions contribute significantly to the binding, involving residues Trp351, Thr516, and Tyr534 (Table 2). These contacts, occurring at distances between 3.69 Å and 3.79 Å, facilitate the positioning of the ligand within the predominantly hydrophobic regions of the active site. Furthermore, an extensive network of hydrogen bonds plays a crucial role in anchoring the ligand. Eight hydrogen bonds were identified involving residues Ser314, Ser368, Asn370, Lys513, Thr516 (engaging both backbone and sidechain atoms), Tyr534, and Tyr566 (Table 3). Notably strong hydrogen bonds, characterized by shorter donor-acceptor distances, were observed with Asn370 (H-A distance 2.26 Å) and Ser368 (H-A distance 2.42 Å). Most hydrogen bonds involved the protein acting as the donor; however, the sidechain oxygen of Tyr534 was predicted to act as an acceptor for a hydrogen bond donated by the ligand UNL. The specific arrangement of these interactions, visualized in the 2D schematic (Fig. 6B), underscores the molecular complementarity between the ligand and the PBP1a binding site.

The 2D schematic **(**Fig. 6B**)** of the ligand–protein interface, showing the spectrum of non-covalent interactions that stabilize binding. Light-green shaded circles denote van der Waals contacts; dark-green dashed lines mark conventional hydrogen bonds; pale-green dashed lines indicate carbon–hydrogen bonds; purple dashed lines show π–sigma interactions; and pink dashed lines represent π–π stacking interactions. Together, these contacts illustrate the diverse interaction network underpinning the ligand’s affinity.

The results from this docking study corroborate the known inhibitory mechanism of amoxicillin, where the antibiotic binds competitively to the transpeptidase active site of PBP1a, effectively disrupting peptidoglycan cross-linking in bacterial cell wall synthesis. The Top binding affinity of − 8.406 kcal/mol suggests a thermodynamically favorable complex formation, supporting the efficacy of amoxicillin in inhibiting bacterial growth. Furthermore, these findings offer insights into the molecular basis of β-lactam resistance, where subtle structural variations in PBP1a may impact the binding affinity of antibiotics.

#### Molecular dynamic simulation and MMPBSA analysis

##### Structural dynamics of the amoxicillin binding to Penicillin-Binding protein 1a (PBP1a) from Staphylococcus aureus complex

The molecular dynamics (MD) simulation of amoxicillin binding to Penicillin-Binding Protein 1a (PBP1a) from *Staphylococcus aureus* reveals key mechanistic insights into this therapeutically relevant interaction. A 100 ns MD trajectory analysis demonstrates that the PBP1a-amoxicillin complex exhibits dynamic stability with localized conformational adaptations that preserve binding specificity without compromising the overall protein architecture.

**Fig. 7:**
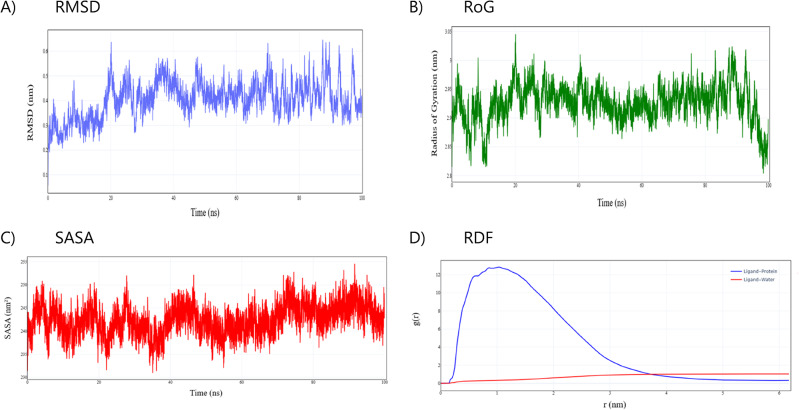
Conformational and structural dynamics of the amoxicillin-PBP1a complex during 100 ns molecular dynamics simulation. Root Mean Square Deviation (RMSD) of protein backbone atoms, revealing a three-phase conformational adaptation process: initial adjustment (0–15 ns), transition with larger fluctuations (15–25 ns), and dynamic equilibrium (25–100 ns) (**A**), Radius of Gyration (Rg) analysis demonstrating maintenance of overall structural integrity (2.8–3.05 nm range) despite localized conformational changes (**B**), Solvent Accessible Surface Area (SASA) measurements reveal constrained fluctuations in the range of (~ 230.9–254.6) nm² with fluctuations correlating with conformational events (**C**), Radial Distribution Function g(r) comparing amoxicillin-PBP1a (blue) and amoxicillin-water (red) interactions, demonstrating strong preferential binding to protein residues at ~ 1 nm (g(r) ≈ 12.8) with minimal solvent interaction (**D**).

RMSD analysis (Fig. 7A) reveals a distinct multi-phase conformational adaptation process. In the initial phase (0–15 ns), PBP1a undergoes structural adjustment as RMSD values increase from ~ 0.06 nm to ~ 0.48 nm (Mean ± SD: 0.301 ± 0.045 nm), reflecting the protein’s accommodation of the antibiotic. A subsequent transition phase (15–25 ns) shows heightened fluctuations, with RMSD ranging from ~ 0.24 to ~ 0.64 nm (Mean ± SD: 0.401 ± 0.071 nm). The final equilibrium phase (25–100 ns) displays stabilized but flexible dynamics, with RMSD values oscillating between ~ 0.27 and 0.65 nm (Mean ± SD: 0.428 ± 0.059 nm; Median: 0.423 nm). Excursions occur around 60 ns (~ 0.52 nm) and 80–90 ns (~ 0.65 nm). These results support the concept of protein-ligand interactions occurring within dynamic conformational ensembles, where transient conformations may represent functionally relevant binding-competent states—a critical consideration in rational drug design.

Rg analysis (Fig. 7B) confirms the preservation of PBP1a’s overall structural integrity throughout the simulation. Rg values remain within a narrow range of ~ 2.80 to ~ 3.05 nm (Mean ± SD: 2.926 ± 0.032 nm). Temporal correlation with RMSD peaks—such as at 20 ns (Rg ~ 2.99 nm)—suggests that localized expansions support functional domain reorientation rather than global unfolding. These findings indicate that amoxicillin induces selective conformational changes at the binding site, maintaining protein compactness and integrity.

SASA measurements (Fig. 7C) reveal constrained fluctuations in the range of ~ 230.9–254.6 nm² (Mean ± SD: 242.5 ± 3.2 nm²), indicating consistent solvent exposure of the complex. Specific SASA increases around 20 ns (~ 240.7 nm²), 40 ns (~ 242.5 nm²), and 80 ns (~ 245.5 nm²) coincide with RMSD fluctuations, supporting the view that internal conformational changes subtly influence solvent accessibility. The relative constancy of SASA suggests that the binding pocket remains well-shielded from bulk solvent, reinforcing the notion of a stable, functionally optimized binding site.

RDF analysis (Fig. 7D) quantitatively affirms the existence of specific interactions between amoxicillin and PBP1a. The ligand-protein RDF (blue curve) displays a sharp peak at *r* ≈ 1.04 nm with g(r) ≈ 12.8 and an approximate FWHM of ~ 0.02 nm, indicative of a well-defined and preferred interaction distance. In contrast, the ligand-water RDF (red curve) shows much lower density (max g(r) ~ 0.33) within the same range, confirming minimal solvent interference at the primary binding site. The crossover point, where water interactions become more probable than protein interactions (g(r) > 1), occurs at *r* ≈ 3.74 nm. This distance threshold offers a benchmark for assessing the solvent exclusivity and depth of novel ligands within the PBP1a binding pocket.

##### Hydrogen bond interactions and residue flexibility in the amoxicillin to Penicillin-Binding protein 1a (PBP1a) complex

**Fig. 8:**
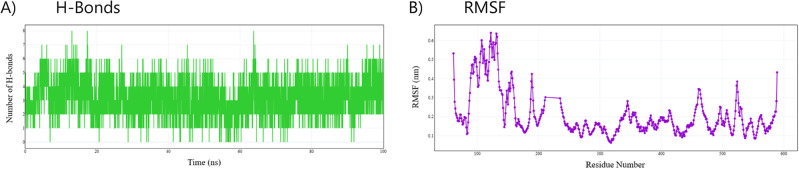
Molecular dynamics analysis of amoxicillin-PBP1a interactions in Staphylococcus aureus. The time evolution of hydrogen bond formation between amoxicillin and PBP1a over a 100 ns simulation period is shown in panel (**A**). The y-axis represents the number of hydrogen bonds, ranging from 0 to 8, while the x-axis shows the simulation time in nanoseconds. Panel (**B**) displays the Root Mean Square Fluctuation (RMSF) profile of PBP1a residues when complexed with amoxicillin. The y-axis shows RMSF values in nanometers, indicating the flexibility of each residue, while the x-axis represents the residue number from approximately 50 to 600.

The molecular dynamics simulation provides insights into the binding mechanism of amoxicillin to Penicillin-Binding Protein 1a (PBP1a) in *Staphylococcus aureus*. As shown in Fig. 8A, hydrogen bond analysis revealed a dynamic interaction pattern throughout the 100 ns simulation. The number of hydrogen bonds fluctuated, potentially ranging from 0 to 8, with the majority of the simulation possibly showing 2–5 concurrent hydrogen bonds. This suggests that the amoxicillin-PBP1a complex could be stabilized primarily through multiple transient hydrogen bonds rather than through a single, persistent interaction.

The hydrogen bond analysis would illustrate the dynamic nature of amoxicillin-PBP1a interactions throughout the 100 ns simulation. A plot would reveal fluctuating hydrogen bond counts, potentially ranging between 1 and 7, perhaps with peaks where the system momentarily formed 7–8 hydrogen bonds. These periods of increased interaction likely represent optimal binding conformations that could inform future drug design efforts. The persistent presence of multiple hydrogen bonds throughout most of the simulation would support the binding affinity observed in energetic analysis. Brief periods where hydrogen bonding might drop to zero could indicate temporary dissociation events reflecting the intrinsic flexibility of the binding pocket, yet the rapid reformation of these bonds would demonstrate the overall stability of the complex.

The RMSF analysis presented in Fig. 8B provides insights into the conformational flexibility of PBP1a residues during amoxicillin binding. The profile reveals several regions with increased flexibility. This is apparent in residues 100–150, where RMSF values reached a maximum of 0.641 nm (mean = 0.44 ± 0.13 nm). This flexible region corresponds to a loop structure within the penicillin-binding domain (residues 27–207). Additional flexibility was observed around residues 350–400 (mean = 0.16 ± 0.03 nm, range: 0.114–0.223 nm) and residues 450–500 (mean = 0.20 ± 0.06 nm, range: 0.103–0.345 nm), which fall within the transpeptidase domain responsible for peptidoglycan cross-linking activity. In contrast, residues 200–300 demonstrated lower RMSF values, ranging from 0.092 nm to 0.303 nm (mean = 0.16 ± 0.05 nm), suggesting a more rigid structure that likely contributes to the stability of the protein’s core catalytic domain.

This variable flexibility profile contributes to amoxicillin’s effectiveness by allowing it to maintain contact with PBP1a even during conformational changes that might otherwise disrupt a more rigid binding mode. The flexibility in the penicillin-binding domain loop (residues 100–150) may facilitate initial antibiotic recognition, while the lower RMSF values in portions of the catalytic domain (e.g., residues 200–300) reflect the need to maintain structural stability in regions directly involved in the function that amoxicillin disrupts. The correlation between regions of flexibility and domain boundaries suggests that PBP1a undergoes domain-specific conformational changes upon amoxicillin binding, a factor in determining antibiotic specificity and efficacy.

The intermediate flexibility observed in residues 350–400 and 450–500 within the transpeptidase domain may indicate regions involved in transmitting conformational changes from the binding site to catalytic residues. This allosteric mechanism could be leveraged to develop novel inhibitors that target these communication pathways rather than competing directly with the substrate for the active site. Overall, the hydrogen bond dynamics (as interpreted originally) and the updated residue flexibility analysis provide complementary evidence for the mechanism of amoxicillin binding and inhibition of PBP1a function in *Staphylococcus aureus*.

##### Penicillin-Binding protein 1a (PBP1a) secondary structure

**Fig. 9:**
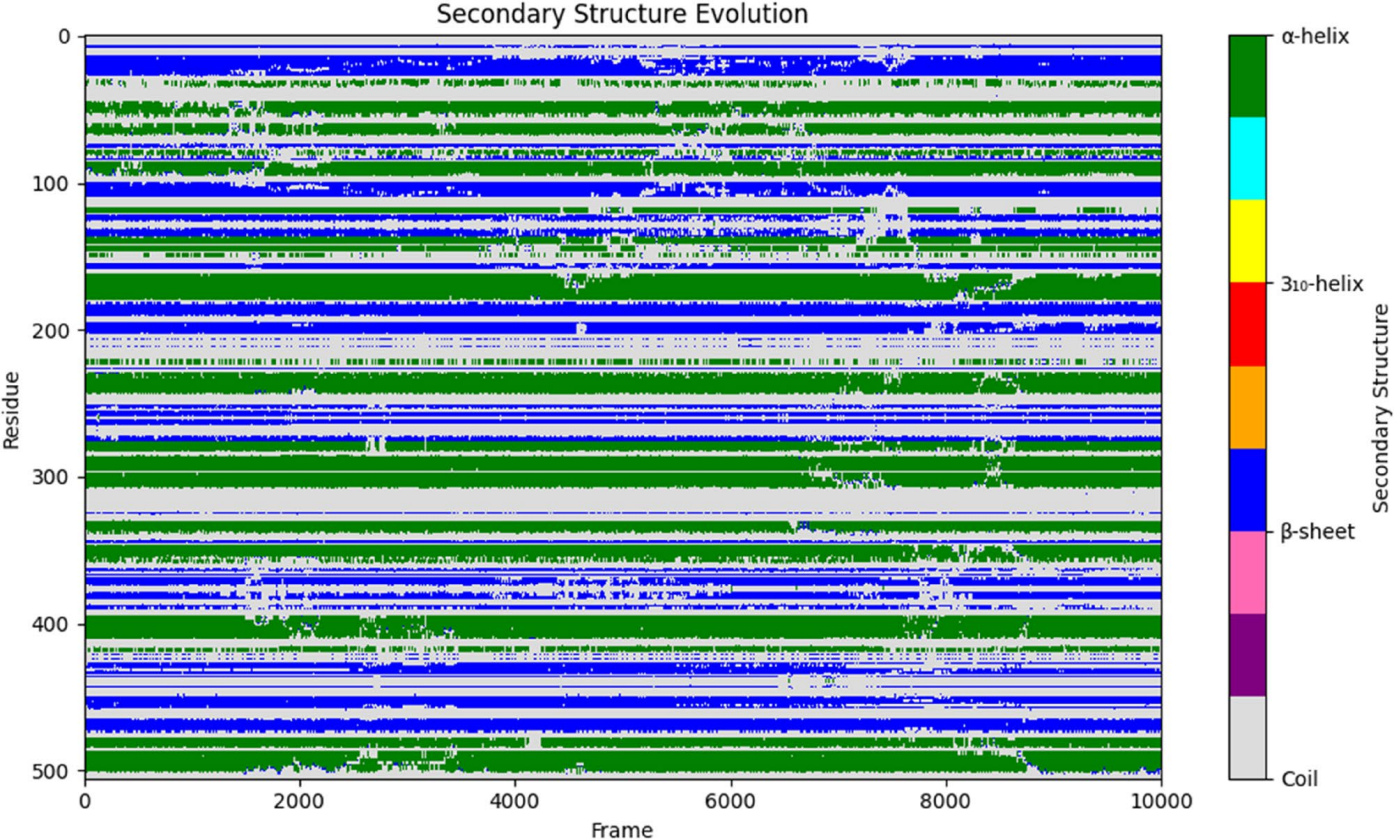
Evolution of PBP1a secondary structure during 100 ns MD simulation complexed with amoxicillin. The y-axis represents the protein residue index, and the x-axis represents the simulation frame number (0−10000, corresponding to 0−100 ns). Secondary structure types are color-coded as indicated in the legend: α-helix (green), β-sheet (blue), 3₁₀-helix (yellow), and Coil (grey/white) are the most represented. The plot illustrates the overall stability of major secondary structural elements throughout the simulation.

The stability of Penicillin-Binding Protein 1a (PBP1a) secondary structure elements upon binding with amoxicillin was assessed over the 100 ns molecular dynamics simulation trajectory. Analysis of the secondary structure evolution across all residues (Fig. 9) revealed that the protein largely maintained its structural integrity throughout the simulation period. Quantitative assessment of the secondary structure content across the entire trajectory indicated a predominance of coil structures (C), accounting for 46.92% of the residues’ conformational states. Alpha-helical structures (H) represented the second most abundant secondary structure element, comprising 31.11% of the conformations, while beta-sheets (E) constituted 21.98%. The visual representation in Fig. 9 illustrates that major helical and sheet regions, particularly those constituting the core domains like the transpeptidase domain, remained largely stable, exhibiting minimal transitions to coil states. Some fluctuations were observed, primarily in loop regions or at the termini of secondary structure elements, consistent with the inherent flexibility observed in the RMSF analysis (Fig. 8B). This overall stability of the secondary structural elements, especially the significant proportion of conserved alpha-helices and beta-sheets within the functional domains, suggests that amoxicillin binding does not induce large-scale unfolding but rather stabilizes a specific conformational ensemble necessary for its inhibitory action, consistent with the persistent interactions observed in docking and hydrogen bond analyses.

##### Energetic component analysis of the amoxicillin-PBP1a complex

**Fig. 10:**
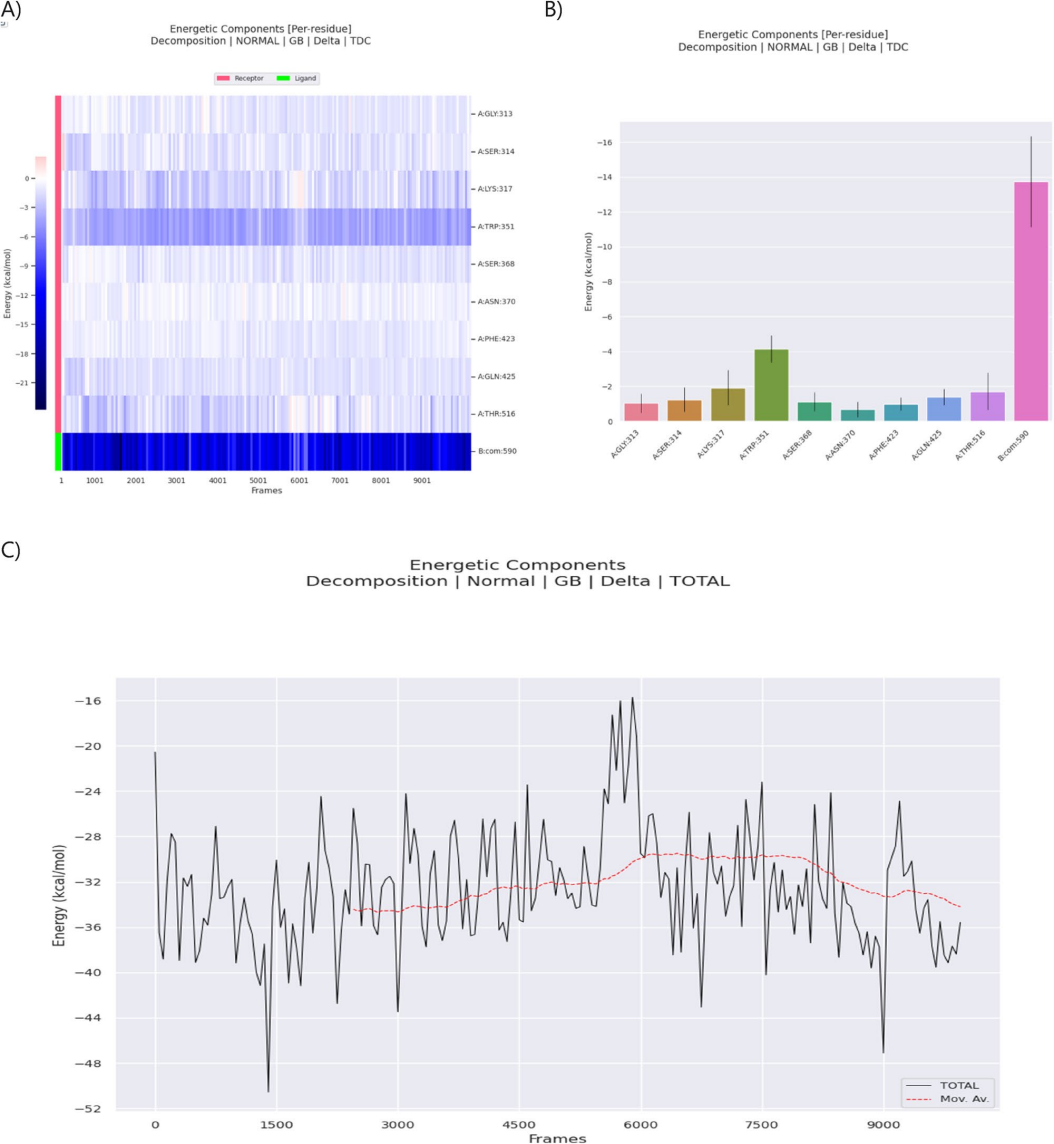
Illustrates the energetic components analysis of the amoxicillin-PBP1a complex in *Staphylococcus aureus* derived from 200 simulation frames. (**A**) Residue-specific heatmap of energetic contributions, distinguishing between receptor (pink) and ligand (green) residues, with darker blue indicating more negative (favorable) energetic contributions. (**B**) Quantitative bar graph of mean per-residue energy contributions averaged over the 200 frames, with error bars representing standard deviation. (**C**) Temporal evolution of total binding energy across the simulation snapshots, demonstrating the net interaction strength between amoxicillin and PBP1a (Average ΔTOTAL =−32.65 kcal/mol, SD = 5.17 kcal/mol).

The energetic decomposition of the amoxicillin–PBP1a complex in *Staphylococcus aureus*, shown in Fig. 10, offers a detailed molecular view of how this essential β-lactam antibiotic interacts with its enzymatic target. The analysis, derived from 200 complex frames extracted from a molecular dynamics (MD) simulation trajectory, captures the interactions as amoxicillin interacts with PBP1a. By examining both residue-specific energetic contributions and the overall binding energy, this approach sheds light on the residues that stabilize the antibiotic in the active site and the overall binding strength.

In Fig. 10A, a residue-specific heat map shows the energetic contributions of the receptor (PBP1a) and ligand (amoxicillin), where darker blue areas indicate more negative, or favorable, interactions. The β-lactam core of amoxicillin, represented in green, is particularly significant, with substantial negative energy contributions indicating its central role in the binding process. This is consistent with the well-established role of β-lactams in docking into the catalytic site of penicillin-binding proteins, positioning the β-lactam ring for nucleophilic attack. The consistently negative energetic contributions from the antibiotic scaffold suggest strong enthalpic interactions, such as van der Waals forces and hydrogen bonding, which collectively secure amoxicillin in the active site of PBP1a.

Figure 10B further explores the binding interface by presenting a bar graph of mean per-residue energy contributions averaged over the 200 frames, with error bars representing the standard deviation. Residues such as A: THR:516 exhibit a notable negative energy contribution (Average TOTAL:−1.71 kcal/mol), indicating a significant role in stabilizing the complex, likely engaging amoxicillin through hydrogen bonds and anchoring the ligand. A: PHE:423 also shows a favorable negative energy contribution (Average TOTAL:−0.99 kcal/mol), highlighting the role of aromatic residues in organizing and stabilizing the antibiotic within the active site. A: TYR:566 contributes less significantly to the direct binding energy (Average TOTAL:−0.50 kcal/mol), suggesting its role might be more involved in structural positioning or other interactions not fully captured by this decomposition term alone. Contrary to the initial interpretation, A:SER:314 shows a favorable energetic contribution (Average TOTAL:−1.25 kcal/mol), suggesting it contributes to stabilizing the ligand rather than having a near-neutral role. These analyses emphasize the dynamic nature of ligand binding captured by MD simulations.

The energetic analysis of the amoxicillin-PBP1a complex reveals additional key residues. A: TRP:351 exhibits a substantial negative energy contribution of approximately−4.15 kcal/mol (Average TOTAL), indicating it serves as a significant stabilizing force in the complex. This tryptophan residue likely engages in favorable van der Waals and potentially electrostatic interactions with amoxicillin’s ring structures. These interactions help position the antibiotic optimally within the binding pocket. The consistent negative energy contribution suggests A: TRP:351 maintains stabilizing interactions, anchoring amoxicillin.

A: GLN:425 (note: residue is Glutamine (GLN) in the data) also presents a negative energy contribution of approximately−1.39 kcal/mol (Average TOTAL), indicating a favorable interaction with amoxicillin. This glutamine residue likely forms hydrogen bonds or engages in other electrostatic interactions that help secure the antibiotic in the binding pocket.

Figure 10C displays the temporal evolution of the total binding energy. While the specific range across frames isn’t detailed in the summary files, the average total binding energy over the 200 frames is consistently negative at−32.65 kcal/mol, with a standard deviation of 5.17 kcal/mol. This confirms the strong affinity of amoxicillin for PBP1a, with fluctuations indicating structural rearrangements and dynamic interactions within the complex. These energy changes may correspond to transitions between different binding conformations, in line with the concept of induced-fit recognition. The variation in binding energy highlights minor conformational adjustments reflective of local adaptations in the binding site or slight shifts in ligand orientation.

Table 3 presents the average free binding energies (ΔTOTAL) and individual energy terms for the amoxicillin-PBP1a complex across the 200 simulation frames. The average values for van der Waals energy (ΔVDWAALS:−37.82 kcal/mol) and electrostatic energy (ΔEEL:−46.91 kcal/mol) indicate highly favorable binding interactions. The non-polar contribution to solvation energy (ΔESURF:−5.76 kcal/mol) also favors binding. The positive value for the polar solvation energy (ΔEGB: 57.84 kcal/mol) largely reflects the desolvation penalty upon binding, which is typical. The total Gas phase energy (ΔGGAS = ΔVDWAALS + ΔEEL) is−84.73 kcal/mol, and the total Solvation free energy (ΔGSOLV = ΔEGB + ΔESURF) is 52.08 kcal/mol. The total binding free energy (ΔTOTAL), averaging−32.65 kcal/mol, indicates a strong overall affinity between amoxicillin and PBP1a. The standard deviations (SD) for these values, particularly in electrostatic (SD: 8.50 kcal/mol) and polar solvation (SD: 6.69 kcal/mol) energies, reflect the dynamic nature of these interactions over the simulation.

**Table 3:** Average free binding energies (ΔTOTAL) and individual energy components for the amoxicillin–PBP1a complex calculated over 200 simulation frames.

Energy Component	ΔVDWAALS	ΔEEL	ΔGGAS	ΔEGB	ΔESURF	ΔGSOLV	ΔTOTAL
Average Value (kcal/mol)	−37.82	−46.91	**−84.73**	57.84	−5.76	**52.08**	**−32.65**
Standard Deviation (SD, kcal/mol)	3.37	8.50	**9.39**	6.69	0.34	**6.56**	**5.17**

Mechanistically, these findings align with the established role of β-lactam antibiotics as “suicide substrates.” Initially, the ligand adopts a noncovalent pose in the catalytic cleft of PBP1a, aligning the β-lactam ring for nucleophilic attack on the active-site serine. This alignment results in the formation of a covalent acyl-enzyme intermediate, which inactivates the protein. The strong negative binding energy (−32.65 kcal/mol) reflects a stable pre-covalent state, where the β-lactam ring is positioned near the nucleophilic serine, stabilized by the identified key residues through van der Waals and electrostatic interactions. The energy variations observed during the simulation (SD: 5.17 kcal/mol) suggest ongoing conformational shifts as the system samples intermediate conformations potentially leading towards the acylated state.

## Conclusion

This study successfully synthesized and characterized amoxicillin-conjugated Fe₃O₄@SiO₂ magnetic nanoparticles (Amox-MNPs) and evaluated their potential against MRSA by targeting PBP1a. Our integrated approach combined experimental validation with computational modeling. In vitro assays demonstrated that Amox-MNPs possess significantly enhanced antibacterial activity against S. aureus compared to free amoxicillin, with nearly a two-fold increase in the inhibition zone diameter. This enhancement is likely due to nanoparticle-mediated advantages, including increased local drug concentration at the bacterial surface and potential protection of the antibiotic from enzymatic degradation. Molecular docking and extensive molecular dynamics simulations provided atomistic insights, confirming stable and energetically favorable binding of amoxicillin within the PBP1a active site, reinforcing the feasibility of targeting this alternative PBP. Together, these results provide a strong preclinical proof‑of‑concept for targeting PBP1a in MRSA using Amox‑MNPs; nonetheless, translation toward clinical application will require rigorous follow‑up studies, including comprehensive in vitro cytotoxicity profiling on mammalian cell lines, in vivo efficacy and pharmacokinetic/biodistribution assessments in animal models, and a formal toxicity and risk evaluation to distinguish the safety profiles of the nanoparticle carrier versus the antibiotic conjugate.

## Electronic supplementary material

Below is the link to the electronic supplementary material.

Supplementary Material 1

## Data Availability

No datasets were generated or analysed during the current study.

## Abbreviations

Amox-MNPs: Amoxicillin-Conjugated Magnetic Nanoparticles
ATCC: American Type Culture Collection
CLSI: Clinical and Laboratory Standards Institute
CFU: Colony-Forming Units
DLS: Dynamic Light Scattering
Fe₃O₄: Magnetite (Iron(II, III) Oxide)
MD: Molecular Dynamics
MM-PBSA: Molecular Mechanics Poisson-Boltzmann Surface Area
MRSA: Methicillin-Resistant Staphylococcus aureus
MNPs: Magnetic Nanoparticles
NDDS: Nanoparticle-Based Drug Delivery Systems
PBP1a: Penicillin-Binding Protein 1a
PBP2a: Penicillin-Binding Protein 2a
PDI: Polydispersity Index
RDF: Radial Distribution Function
RMSD: Root-Mean-Square Deviation
RMSF: Root-Mean-Square Fluctuation
Rg: Radius of Gyration
ROS: Reactive Oxygen Species
SASA: Solvent-Accessible Surface Area
SCCmec: Staphylococcal Cassette Chromosome mec
TEOS: Tetraethyl Orthosilicate
TEM: Transmission Electron Microscopy
UV-Vis: Ultraviolet-Visible Spectroscopy
GROMACS: GROningen MAchine for Chemical Simulations
CHARMM: Chemistry at HARvard Macromolecular Mechanics
CGenFF: CHARMM General Force Field
PME: Particle Mesh Ewald (method for electrostatic calculations)
PLIP: Protein-Ligand Interaction Profiler
